# Reacting to the COVID-19 pandemic through digital connectivity with customers: the Italian experience

**DOI:** 10.1007/s43039-021-00031-y

**Published:** 2021-06-06

**Authors:** Marco Bettiol, Mauro Capestro, Eleonora Di Maria, Stefano Micelli

**Affiliations:** 1 Department of Economics and Management, University of Padova, Padova, Italy; 2 Department of Management, Ca’ Foscari University, Venice, Italy

**Keywords:** COVID-19, Digital technologies, Web-based technologies, Industry 4.0, Customer relationships

## Abstract

The COVID-19 has deeply impacted the firm’s competitiveness because of the restrictions that limited the relationships with existing and new customers. The pandemic has pushed firms to rely on digital technologies to redefine business processes as well as customer relationships and marketing strategies. The digital technological portfolio firms may rely on to face the COVID-19 related challenges spanning from the established web-based technologies to the more recent Industry 4.0 tools related to the fourth industrial revolution. In this regard, the paper aims at exploring which digital technologies allowed firms to positively react to the pandemic to overcome their constraints in managing the market relationships. Based on an original qualitative analysis on 26 Italian SMEs carried out during the first Italian lockdown in 2020, the paper identifies three strategies in the use of digital technologies to support customer relationship management and market expansion. It emerges specifically the strategic importance of web-based technologies (videoconferencing, CRM and e-commerce) to support firm competition and performance through customer interactions and digital experience, advancing the literature on firms’ reaction strategies during turbulent and crisis times.

## Introduction

The COVID-19 pandemic had a dramatic impact on several countries across the world. National governments have imposed widespread restrictions to prevent the development of the pandemic (Verma & Gustafsson, 2020). The new competitive scenario induced by the COVID-19 crisis raised many issues on how business activities should be re-organized due to the difficulties of physical interactions with employees, distributors, suppliers and customers (Sheth, 2020). The pandemic has particularly affected the marketing processes because of the relevant issues emerged in managing physical sales channels and interactions with customers, both in the Business-to-Consumer (B2C) and in the Business-to-Business (B2B) markets (Kang et al., 2020; Wang et al., 2020).

Recent research about the appropriate actions and strategies that could help firms to overcome the crisis has highlighted the key role of digital technologies that may ensure connections and, thus, help business activities to run smoothly (Yin et al., 2020). This could be true especially with the emergence of strong limitations on physical interactions as during the COVID-19 pandemic (Papadopoulos et al., 2020). In such a scenario, the online channel becomes the most important environment for customers to get in contact with the firm and carry out purchasing activities (Hoekstra & Leeflang, 2020). Firms should push technological investments to provide a digital experience, manage product configuration and/or support sales directly (Siggelkow & Terwiesch, 2019). In this specific environmental condition, different digital technologies may help firms to define innovative strategies to approach the market as well as to define new business models (Chesbrough, 2020), enabling firms to develop new business opportunities (Nenonen & Storbacka, 2020).

Past marketing research has highlighted the potential of information technologies (IT) and specifically of web-based technologies in designing effective customer relationship management both in terms of one-to-one marketing (Peppers et al., 1999) and community-based framework (Armstrong & Hagel, 1996; Kozinets, 1999). According to scholars, the revolution of the web completely transformed marketing strategies (Sharma & Sheth, 2004) as enabled firms to benefit from digital technologies in terms of exchanging information with customers and managing complex relationships to strengthen product innovation, brand management, and more broadly value creation.

More recently, studies on Industry 4.0 technologies have pushed forward this debate by emphasizing the predictive potential of data analytics for marketing (Davenport et al., 2020). The new wave of technologies that firms may use in the production domain, such as 3D printing, connected with data-processing technologies, such as big data, artificial intelligence (AI) and Internet of Things (IoT), all included under the umbrella concept of Industry 4.0 (Frank et al., 2019b), have opened new scenarios for value creation (Sauter et al., 2015) linked to the firm’s possibility to increase data gathering and the analysis of customers’ needs and behaviors, through constant active interactions with customers as well as passive data collection flows (i.e., through smart products) (Iansiti & Lakhani, 2014; Porter & Heppelmann, 2014). Based on such innovative technologies, the firm may redesign their offerings in terms of new products and servitization (Frank et al., 2019a) within a deeper customer relationship management connected to a marketing 4.0 scenario (Kotler et al., 2017).

Despite the attention on the key role of digital transformation in facing the COVID-19 crisis and better positioning after the COVID-19 pandemic (Rapaccini et al., 2020), there is very limited evidence on how the different digital technologies (web-based vs Industry 4.0) supported firms to overcome the challenges emerged from the COVID-19 pandemic. In so doing, the paper aims at exploring the relationship between digital technologies and the firm’s reaction to pandemic challenges, focusing on the context of customer relationship management. More specifically, the goal is to investigate to what extent the mentioned digital technologies have positively supported the firm’s reaction to the pandemic constraints in their market relationships. Based on an original empirical analysis of 26 Italian firms interviewed during the first Italian lockdown in 2020, the paper highlights the key role of web-based technologies to immediately interact with customers and try to overcome the pandemic challenges, advancing the literature on the firms’ reaction strategies to the COVID-19 crisis and, more broadly, on firm’s strategies in turbulent and crisis times. In particular, the paper shows that web-based technologies may be able, in the short time, to put in place new marketing strategies to overcome the crisis.

The paper is structured as follows: in section two we review the literature on customer relationships and digital technologies, taking into consideration also the crisis period; in sections three and four, the methodology and results are presented; in section five we discuss the results and then a final conclusive section is devoted to propose theoretical and managerial implications as well as limitations of the study and suggestions for future research.

## Theoretical background

### Customer relationships and digitalization

The rise of Internet and of the Web has deeply transformed the use of IT with respect to the relationships between the firm and the customers. According to literature (Peppers et al., 1999; Wells et al., 1999), through its own website, the firm is able to create a one-to-one relationship with customers, by enhancing customized communication and using data collection for targeted value propositions. The Web becomes a new bidirectional communication channel where the firm can define multiple contents and where also customers can contribute directly (Sharma & Sheth, 2004). At the same time, through Internet it becomes possible to couple the extended market reach and richness in customer relationships (Evans & Wurster, 2000), opening mass customization opportunities.

The revolution of the Web has also enhanced knowledge management processes related to the customer, empowering customers and reducing their dependence on the firm (Micelli, 2017). Due to the virtual environment and the advent of online communities of consumption (Kozinets, 2002), customers become active players in the process of new product development (Nambisan & Nambisan, 2008) as well as in supporting other customers based on their purchasing experience or specialized knowledge (Sawhney et al., 2005). Online communities are essential for supporting business innovation through open knowledge sharing (Marchi et al., 2011; Schau et al., 2009). According to the digitalization of customer relationship management, marketing processes are enhanced, and marketing strategies can be redesigned to leverage on the proactive role of customers during the product lifecycle and to identifying new value generation opportunities (Siggelkow & Terwiesch, 2019).

The development of e-commerce has in particular further reinforced this intimacy and connection with customers. On the one hand, the product sale and its communication process are tightly connected through the development and management of digital content (Chaffey et al., 2019). Brand management becomes a shared process where customers are directly involved and can participate in nurturing brand meaning through their communication (Kotler et al., 2017). Moreover, customers can provide feedback and the firm can rely on the analysis of data based on past purchasing behavior for recommendation and product development. On the other hand, the e-commerce scenario impacts on the firm’s channels management, where the customer has multiple online entry points to collect information, compare products and to complete her purchasing activities.

The CRM solutions represent an Internet technology that enables the integration among the different processes connected to the customer and the related information (Sin et al., 2005). CRM allows gathering and managing customer information effectively to better understand their needs and strategically support customer lifecycle management in relation with e-commerce as well as social media (Acker et al., 2011; Kotler et al., 2017). This technology is considered essential to build effective relationships with customers for both B2B and B2C firms as allows them “to formulate more appropriate marketing strategies and to execute specific marketing actions more efficiently and quickly by offering superior first-line support and the access of integrated access customer data” (Chang et al., 2010, p. 850). From this perspective, effective customer relationship management (CRM) becomes a strategic imperative for each firm that could be closer to their customers and, thus, they should put more effort in finding new ways to create value from the information they receive, to improve the customer journey as well as her loyalty (Park & Kim, 2003).

This scenario has been recently transformed due to the increased attention for new technologies related to the concept of Industry 4.0. This wide set of technologies has promised to radically transform manufacturing activities (Alcácer & Cruz-Machado, 2019), but also marketing processes. Through advanced and connected automation or additive manufacturing (i.e., 3D printing) firms may increase opportunities for product customization in a mass customization paradigm, up to tailored solutions for customers (Di Roma, 2017; Liao et al., 2017). More specifically, the Industry 4.0 technologies are connected to data management and predictive analytics (Tao et al., 2018) that represent one of the most relevant areas for marketing (Erevelles et al., 2016). In the fourth industrial revolution, firms can invest to transform their products into smart products (IoT) to create a constant data flow enabling further product and service development as well as new sources of value creation (Iansiti & Lakhani, 2014; Porter & Heppelmann, 2014). Specifically, data-processing technologies may represent interesting opportunity marketing-wise. The possibility to analyze customer behavior and to exploit their feedback for new offering is made possible by big data analytics and AI solutions (Davenport et al., 2020; De Luca et al., 2020). There are multiple marketing applications to support the relationships with customers in the AI domain (Grandinetti, 2020; Mandelli, 2018). Firms can exploit big data analytics to provide better customer services, by anticipating customers’ needs and behaviors, also relying on a larger scale of data to provide a more refined and advanced market analysis. Another important related area of AI-supported marketing refers to the customization of online communication strategies designed to optimize the customer’s experience (Brill et al., 2019).

### Managing customers in the pandemic scenario: investigating the role of technologies

The COVID-19 pandemic, and the following crisis emerged, has radically put attention on how firms effectively reach customers, specifically when physical interactions and mobility have been limited by lockdown. Digital technologies have been highlighted as important tools firms can use to overcome the limitations of the pandemic (Soto-Acosta, 2020) as they may enable business processes and services to ensure continuity and interaction while data are gathered to define new strategies (Papadopoulos et al., 2020).

The pandemic has impacted on customer relationship management in multiple directions, both in the B2C and B2B markets. First, firms have experienced limits in interacting with customers during the sales process due to the lockdown, especially when customized products have to be developed. Without the possibility to interact face-to-face firms may have seen a reduced possibility to develop products that requires rich interaction with customers (Wu et al., 2016). Second, lockdown has interrupted retailing activities rooted on physical sales through brick-and-mortar stores, with negative consequences specifically for firms with no or limited ecommerce solutions already in place. Third, the limitation in international mobility has also challenged firms operating in international markets, in terms of information provision to potential customers related to their offerings or the possibility to reach existing customers (Zafari et al., 2020). This is particularly relevant in the B2B context, where often buyers and suppliers meet in person and the communication on products and purchasing process are defined interactively (Österle et al., 2018). Indeed, the COVID-19 pandemic has negatively impacted not only the B2C market (Chesbrough, 2020), but also the B2B context (Cankurtaran & Beverland, 2020) where firms had to react to limitations in sales activities (Hartmann & Lussier, 2020) by redesigning the organizational resources, also enhancing the use of technological infrastructures to manage the progressive blurring between channels. From this point of view, several constraints on national and international mobility have blocked or reduced the possibility of organizing business fairs that were one of the most important marketing initiatives in the B2B context (Kang et al., 2020).

On the one hand, recent reports on COVID-19 have stressed the rise in the use of Internet and social media (Donthu & Gustafsson, 2020). The customer’s behavior has changed during the lockdown and a massive use of the web and social media have become the channels to manage product information gathering and purchasing activities. Especially digital products or priority goods, such as food, have experienced an increased in sales, while many others have suffered. The crisis has pushed firms to overcome the challenges in reaching and managing markets by further considering digitalization within the web-based environment (Kim, 2020; Papadopoulos et al., 2020).

On the other hand, the fourth industrial revolution centered on Industry 4.0 technologies opens new opportunities for enhancing customer relationship management to overcome the challenges mentioned above. Data-processing technologies that rely on data to provide information-driven input for decision making (Culot et al., 2020) can support firms in developing innovative products/services, to approach existing markets or enter in new ones, as well as in defining new business models and supporting the decision-making process, leveraging on data about customers and markets (Frank et al., 2019a).

The COVID-19 crisis has been particularly rapid in its spread across industries, markets, and countries, requiring firms of adopting solutions very quickly to not suffer from the negative consequences of lockdown (Eggers, 2020). This scenario opens new issues on the technologies adopted (He et al., 2021) and on the marketing processes (Kang et al., 2020) supported by such digitalization during the pandemic, specifically as far as customer relationship management is concerned. Research on digital transformation highlights that technologies have different characteristics in terms of potentiality for marketing purposes and in terms of time required for implementation (Robert et al., 2020). Previous studies show that both the adoption rate and the implementation process of the different digital technologies (the more mature web-based technologies and the more recent Industry 4.0 technologies) is uneven among firms of different sizes and industries (Bettiol et al., 2020a). It should not be taken for granted that technologies related to the web-based revolution and those of the fourth industrial revolution have been adopted and exploited by firms in the same manner (Ghobakhloo & Fathi, 2020).

Further knowledge is, therefore, required to better disentangle the role of the different digital technologies in supporting firms during the COVID-19 pandemic, specifically in implementing customer-wise marketing activities. This study aims at answering to the following research questions: *Which are the digital technologies that firms mainly used during the COVID-19 pandemic? How did firms use digital technologies to manage customer relationships and react to the COVID-19 challenges?*

## Methodology

The study draws on in-depth interviews carried out on Italian manufacturing small- and medium-sized firms^1^ (SMEs) collected during the COVID-19 pandemic (May and June 2020) to explore how firms responded to the COVID-19 challenges with a main attention on the use of digital technologies and the interaction with customers. Due to the extraordinary event representing our empirical context (the pandemic), following recent literature, we chose an opportunistic method (Burd, 2019) for case study selection. We engaged a sample of Italian SMEs interviewed through an online survey between September 2019 and January 2020, thus immediately before the beginning of the pandemic. The online survey, aimed at assessing the firm’s technological (IT and Industry 4.0 technologies) investment strategy, has been carried out on a stratified sample of Italian SMEs operating in some of the most important Italian manufacturing (such as food, automotive, chemical and pharmaceuticals, mechanics, home systems, and fashion) and services industries (such as logistics and transport and IT) and allowed to gather 366 questionnaires. Findings of the first quantitative study allowed us to set up and deploy the qualitative study related to the COVID-19. The choice to consider the sample of firms involved in the online survey for the interviews carried out during the COVID-19 pandemic was driven by the opportunity to have data about the adoption of different technologies that may favor the exploration about how firms have adapted their strategies in terms of use to face the COVID-19 challenges, especially as a consequence of the changes occurred with customers.

During the first Italian lockdown (April 2020), we engaged by emailing all the firms that participated to the previous online survey (366 firms), for a qualitative study based on in-depth interviews, and we were able to collect 26 useful interviews with firms operating in different industries and markets (B2B and B2C) (Table 1). We interviewed entrepreneurs, CEOs, and/or managers of the firms through a set of open-ended questions that followed an interview guideline in order to keep the conversation structured and on point. Specifically, after a brief description of the firm’s activity, the questions aimed to assess more in depth: (1) the impacts of COVID-19 on business processes and performance as well as on customer interactions; (2) the role of digital technologies in facing the COVID-19 pandemic; (3) the related strategies in terms of process and product innovation (with a focus on customer relationships). For each one of the three key-points investigated, we asked to the respondents to describe the current situation (also in relation to the past) as well as to provide their perception about the future.

**Table 1:** Firms interviewed Source: Author’s elaboration

Firm	Industry	Business activity	Market	Interviewee position
Firm 1	Home system	Production of wooden windows and doors	B2B/B2C	Co-owner/founder
Firm 2	Manufacture of stationery	Production of luxury pen	B2C	CEO
Firm 3	Mechanics	Industrial washing machine for vehicles	B2B	CEO
Firm 4	Consulting	Information technology consulting/services	B2B	IT Manager
Firm 5	Technology	Web-agency	B2B	General Manager
Firm 6	Information technology	Online information and communication services	B2B	CEO
Firm 7	Footwear	Production of woman shoes	B2C	Founder
Firm 8	Information technology	Integrated telecommunication services	B2B	CEO
Firm 9	Home system	Electrical, plumbing and sanitary installations	B2C	Co-owner
Firm 10	Transport	Transport and logistics solutions and national and international shipping	B2B	CEO
Firm 11	Consulting	Certification and consulting services	B2B	CEO
Firm 12	Information technology	Advanced ICT consulting services	B2B	Marketing Manager
Firm 13	Food	Semi-finished products for bakery	B2B	Sales/Marketing Manager
Firm 14	Chemical and pharmaceutical	Production and packaging of medical devices and cosmetics	B2B	CEO
Firm 15	Technology	Software development	B2B	Accounting Manager
Firm 16	Technology	System integration services	B2B	Project Manager
Firm 17	Constructions	Sheet metal working	B2B	Co-owner/founder
Firm 18	Mechanics	Production of filter presses and water treatment plants	B2B	CEO
Firm 19	Furniture	Production of furniture	B2B/B2C	Marketing Manager
Firm 20	Packaging	Production of packaging film	B2B	Financial Manager
Firm 21	Home system	Production of interior doors	B2B/B2C	Co-owner/founder
Firm 22	Jewellery	Production of jewellery	B2C	CEO
Firm 23	Food	Wine production	B2B/B2C	Founder
Firm 24	Mechanics	Design and production of machines and robots	B2B	CEO
Firm 25	Mechanics	Design and production of trailers and semi-trailers	B2B	Accounting Manager
Firm 26	Home system	Production of wooden and aluminium windows and doors	B2B/B2C	Sales Manager

Each interview lasted from 60 to 90 min and benefited from documentation and results from the prior study. All interviews were taped and transcribed verbatim to increase the findings’ reliability (Yin, 2004). To reach our research purposes, from the interviews collected, we proceed through a multiple-process analysis based on: (1) a word cloud analysis to extract the main words emerged from the interview with the firms and summarized through the word cloud; (2) a cross-case analysis based on frequency and content analysis to assess the firm’s technological endowment, its use and main strategic purposes during the pandemic and outline preliminary results; (3) a selected case study analysis (Siggelkow, 2007) to highlight how firms responded to the COVID-19 pandemic based on the results of phase 2.

The interviews collected were processed with the MAXQDA software (released by VERBI GmbH). Starting from the punctual transcription of the interviews conducted, we proceeded to clean the same interviews and relative files (avoiding the redundant question–answer effect). After that, the text of all interviews were loaded into MAXQDA to perform a first textual cleaning (i.e., a list of words to be excluded from the textual analysis such as articles, prepositions and some other words closely related to the pandemic crisis and not pertaining to the research context). Then, the further textual cleaning occurred according to the following criteria: (1) the same word present in both singular and plural was counted as a single word; (2) words that had the same meaning (therefore synonyms) were considered as a single word in the final count; (3) words that take on a different meaning if used in conjunction with other words (typical example, the unemployment benefit fund and smart working) were searched individually in the text of the interviews to understand if they went alone or accompanied by other words. Finally, for the word cloud analysis, after the cleaning process, from the total number of words that emerged, only those with a minimum frequency of 10 were included in the word cloud. In total, 69 words, whose frequency ranges from 10 to 192 formed the cloud. Regarding to the visualization of the words, the following visualization modes made available by the MAXQDA software were chosen: (1) frequency: words more frequent are larger; (2) scale (affects the size differences between common and rare words): larger for more frequent, i.e. the font size is proportional to the square of the word frequency; (3) word-order: random.

In the second step of the analysis, through the frequency analysis, we aimed at assessing the technological endowment of the firms interviewed, taking into consideration information and web-based technologies, such as website, social media and e-commerce (Abed et al., 2015), as well as the enterprise-business management technologies, such as Enterprise Resource Planning (ERP), CRM and Supply Chain Management (SCM) (Hendricks et al. 2007), and the main enabling Industry 4.0 technologies (Frank et al., 2019b). In addition, in this step we proceed to evaluate how the firms interviewed used technologies to face the crisis emerged from the COVID-19 pandemic during the Italian lockdown.

Finally, in the third step of the analysis, taking into consideration both the research purposes and the results emerged from the previous step of analysis, we selected and focused on the three most important case studies (Siggelkow, 2007) to better present how and which main technologies were used to interact with customers and face the market turbulence created by the pandemic in the short-time.

## Results

### Firm’s priorities during the COVID-19 pandemic: a word cloud analysis

**Fig. 1:**
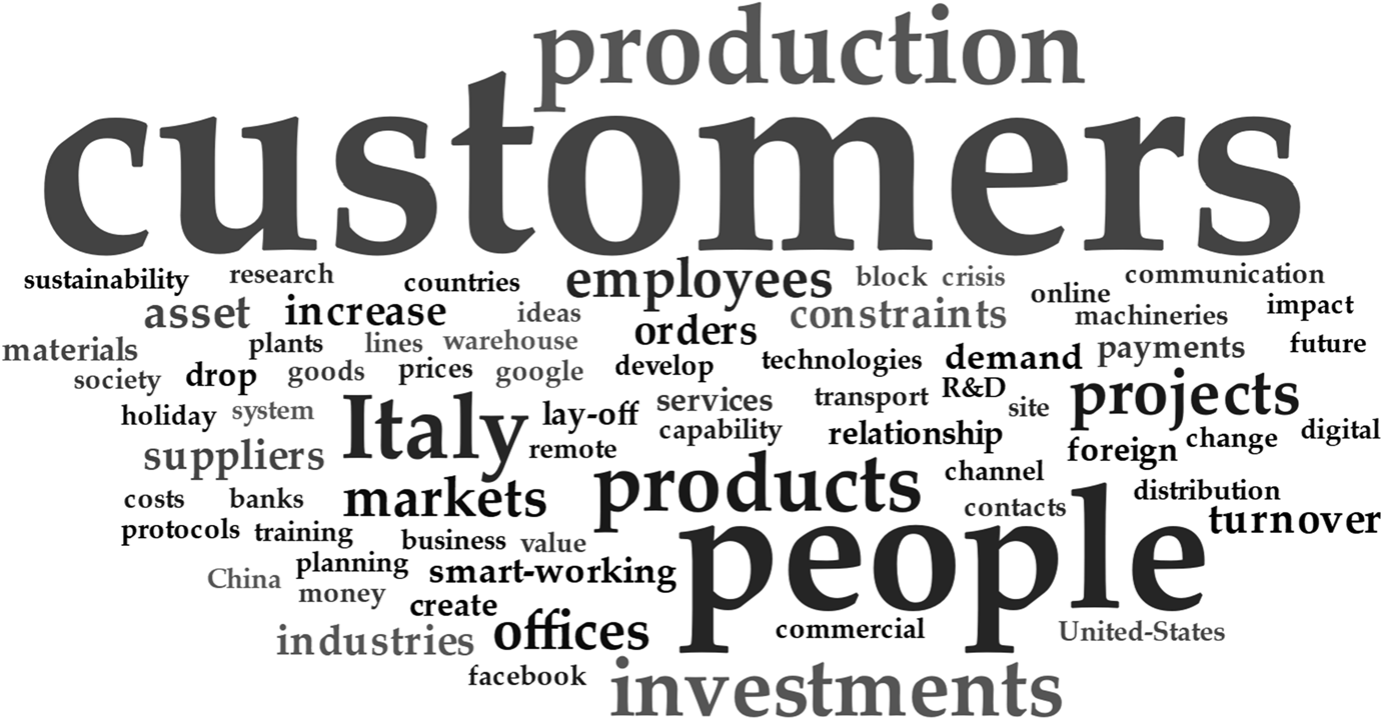
Key words mentioned by interviewed firms Source: Author’s elaboration

The first step of analysis regards the assessment of the key words of the interviews collected to highlight what are the relevant factors related to the research purposes. The word cloud (Fig. 1) is a visual representation of word frequency (Zikmund, 2013) which provides a first quick look for formative purposes (Depaolo and Wilkinson, 2014).

The main result emerged from this first analysis is the strategic role of customers for firms during the pandemic. Indeed, this perspective has been outlined by many firms such as the CEO of Firm 2, who stressed the relevance to take care of customers during the pandemic to assure long-term relationships:

“*We delivered a bar of chocolate with the shape of a nib to all our customers, so it was made as a nib to offer them a start, a little sweeter and a little less bitter than all problems that surely everyone will encounter in the reopening, in the reorganization, of their store, etc. There is the thought of continuing to say and tell that you are there, that you are close and talk to your customers in a different way, and I think this will be a new way to express proximity, the proximity of our brand, the proximity of the product, the proximity of the people, ok technology but also humanity*”.

In addition to the key role of customers, the word cloud reveals other key terms, mainly related to product and production, which need specific consideration due to the restrictions imposed by the pandemic situation. Other key concepts are related to the markets and to Italy (in terms of upstream and downstream strategies) and the investment in new technologies to digitalize the business process with the aim to favor the relationship with customers. These preliminary results allow us to take knowledge about the main factor (customers) on which should be focused the firm strategy to face the COVID-19 pandemic, better explored in the next steps.

### Use of digital technologies during the COVID-19 pandemic

In the second step of the analysis, we firstly assessed the firm’s technological endowment through the data collected by the online survey, and then we focused on assessing, through the content analysis of interviews, the use of digital technologies during the COVID-19 pandemic. As shown by the frequencies reported in Table 2, there is an uneven distribution of technologies adopted, being website, social media, ERP and CRM the most adopted ones (Table 2).

**Table 2:** Technology adoption rate. Source: Author’s elaboration

Technology	Frequency	Percentage (%)
Website	26	100.0
Social media	18	69.2
ERP	16	61.5
CRM	12	46.2
Cloud	8	30.8
E-commerce	4	15.4
SCM	4	15.4
Autonomous robots	4	15.4
Value chain integration systems	3	11.5
Cybersecurity	3	11.5
Artificial intelligence	3	11.5
IoT	3	11.5
3D printing	2	7.7
Big data	2	7.7
Blockchain	1	3.8

The most adopted technologies are web-based technologies such as website and social media that firms mainly use to communicate with the external environment, and ERP and CRM, essential to manage effectively the business processes and relationships with customers. More limited adoption refers to cloud, e-commerce, SCM and autonomous robotics. E-commerce is a strategic technology for SMEs to have access to global markets and to gain competitive advantages (Drew, 2003). SCM and robotics are key technologies production processes (Hofmann et al., 2019). Cloud is a cross-function technology that firms use to storage data (Marston et al., 2011). Consistently with other studies, less adopted technologies can be included into the Industry 4.0 framework, which usually require more complex implementation processes (Frank et al., 2019b).

As far as the use of digital technologies during the COVID-19 is concerned, from the content analysis performed, some interesting findings emerged. In particular, with the interviews, we aimed at assessing if firms have increased the use of digital technologies, or modified the strategic approach to the use of digital technologies to sustain customer relationships and business. Table 3 summarizes the results of the analysis, highlighting the marketing-related processes operated through the use of digital technologies during the COVID-19 pandemic. Specifically, Table 3 points out which digital technologies the firms interviewed used to interact with customers, manage relationships with them, and manage and/or support the sales activities during the COVID-19 pandemic.

**Table 3:** Use of digital technologies during the Italian lockdown. Source: Author’s elaboration

Firm	Digital technologies	Marketing-related process
Firm 1	CRM	Customer management
Firm 2	CRM	Managing sales channels (sales agents, shops)
Firm 3	Videoconferencing (i.e. WeChat with Chinese clients)	Managing distant B2B customers
Firm 4	Open source platform; remote marketing solutions; AI	Customer management and remote services
Firm 5	Google suite for videoconferencing; Workplace; website and social media	Customer interaction and management
Firm 6	Videoconferencing (Skype; Slack; Meet; WhatsApp)	Customer interaction
Firm 7	Newsletter and WhatsApp Improved e-commerce solutions	Customer management Managing sales for B2C segment
Firm 8	Videoconferencing (Meet)	Customer interaction
Firm 9	Emailing	Customer management
Firm 10	Videoconferencing (Skype; WhatsApp; WeChat)	Customer interaction
Firm 11	Videoconferencing; Digitalization of internal processes; improvement of newsletter through CRM; Remote access to information (cloud)	Customer interaction and management Sales management/support
Firm 12	Meet and Teams for videoconferencing; remote access	Customer interaction Customer service provision
Firm 13	Improved e-commerce solutions for the B2C segment	Managing sales for the B2C segment
Firm 14	Videoconferencing	Customer interaction
Firm 15	Videoconferencing with customers; Project management system	Customer interaction and management
Firm 16	Videoconferencing with customers	Customer interaction
Firm 17	Videoconferencing	Customer interaction
Firm 18	Videoconferencing with customers (Meet; Zoom)	Customer interaction
Firm 19	Augmented reality app Improved e-commerce solutions for the B2C segment	Customer engagement Managing sales for the B2C segment
Firm 20	Videoconferencing (WeChat for Chinese clients); Social media; Cloud computing	Customer interaction and management
Firm 21	Videoconferencing Newsletter	Customer interaction
Firm 22	Remote access to company’s ICT infrastructure	Customer management
Firm 23	Videoconferencing (Skype; WhatsApp)	Customer interaction
Firm 24	CRM	Customer management
Firm 25	Videoconferencing; website; social media	Customer interaction
Firm 26	Videoconferencing	Interaction with professionals as intermediaries for sales

From this preliminary overview, a cross-case analysis has been conducted to outline any common paths in the use of digital technologies and overcome the challenges of the pandemic. Table 4 presents a summary depicting the relationships between the most used technologies during the lockdown, the specific use within the relationship with the customer and the broader strategic purpose achieved.

**Table 4:** Main digital technologies used for marketing purposes during the Italian lockdown. Source: Author’s elaboration

Most used technology	Use	Main marketing purpose
Videoconferencing	Interacting with customers	Managing relationship with customers during the pandemic
CRM	Managing customers’ requests or needs effectively	Developing new and customized services
E-commerce	Managing sales with existing customers and new ones	Exploring new market segments (from B2B to B2C)

From the content analysis of the interviews collected, it emerges that firms modified their use of digital technology in terms of both breadth (number of technologies used, i.e., CRM in addition to ERP) and depth (the intensity of use of the single technology, i.e. videoconferencing). Not only firms invested in new technologies that they seldom used before pandemic, but they also increased the intensity of use of the technologies they already have.

For example, the use of videoconferencing technologies to keep in contact with customers and, indirectly, to improve sales performance has been extremely relevant. Firms declared that such technology emerged as a consequence of the physical restrictions imposed by the pandemic. Most of them never used this technology to interact with customers or clients before, but thanks to the pandemic they have been able to manage relationships with existing customers and in the future they will continue to use it, especially for the first preliminary interactions (with new customers). The CEO of Firm 18 maintained:

“*...in 25 years I did ten conference calls with clients, now there are three ones per day. Even with employees we used to wait for them to come in for a meeting, now we do the same through Meet, Zoom and it works just as well, it is not 100% the same thing but you have to get used to it*”.

CRM emerges as another key technology to manage interactions with customers. During the COVID-19 pandemic, firms used such technology not only to effectively manage customer relationships but also to develop new customized services with positive effects on performance. As the CEO of Firm 8 stated:

“*So a few years ago we thought that by putting money on the table for a CRM software we would be okay in reality, we have established a continuous improvement approach and we are still investing in this technology. For example, yesterday we were in a videoconference with 8 people, 4 of which were from our structure, to refine the ticketing management software that is absolutely working, but we always want more*”.

Finally, there is one more technology very useful to face the crisis and overcome the limitations of the lockdown: e-commerce firms invested in to improve the B2C segment. Through such digital strategy, firms have been able to diversify their offer aiming at reaching different customer segments and markets enlarging the firm strategic vision. The Sales Manager of Firm 13 told us:

“*During the lockdown, we enhanced our website and online shop with new features. We did this by enlarging our warehouse and using an improved ERP integration*”*.*

### Managing customer relationships through digital technologies during the pandemic: evidence from three case studies

#### Videoconferencing: the case of Firm 3

Firm 3 specializes in designing and building washing machines for trucks, buses and trains and electric vehicles for internal handling and on-road loads. Firm 3 has a turnover of 9 million € with an export of 50% of the turnover and 36 employees. The company is based in a small town south of Florence (Italy). Operating in both the mechanical and electrical sectors, the company has always been known for its ability to continually improve present products or design new solutions for customer-specific applications.

Although the company has been active during the first lockdown of March and April 2020 (as logistics was one of the industries allowed to operate by Italian law), the company declared that the turnover may have decreased by 35% in the Italian market and 25% at the international level. The main problem came from the bus industry: the reduced mobility and the dramatic stop of the touristic industry has decreased the orders by bus service companies. The railway industry is more stable and the company did not expect a decrease in terms of orders. Indeed, the train company asked for new services related to the disinfection of the cars and Firm 3 developed an ozone machine in collaboration with one of its subcontractors. Regardless the pandemic and lockdown, the company did continue in investing in new products, in particular in the development of new small electric vehicles for the transportation of people in historical centers and of goods within the factory.

One of the most important impacts of the pandemic was the implementation of videoconferencing, especially with distant clients. As the CEO of the company told us:

“*Working abroad, we travel a lot, including China where we export machines for the railway industry. There we have set up video conferences*”.

Although the company has just 36 employees and the internal communication is mainly face to face, Firm 3 was forced to react and to heavily use digital technology and often more than one at the same time. As the company declared:

“*Advanced technologies are used for contact with customers, and they vary across countries. Example for China we use WeCHAT*”.

In the past, international fairs and business travels where the most important communication channels with clients. The pandemic and the use of videoconferencing have changed for good relationships with clients. Unexpectedly, for the company:

“*Using this system has allowed us to increase the frequency of meetings and induce everyone to participate: individual interventions increase with video conferences*”.

This transformation helped the company to increase the quality of service and in particular the process of product personalization that is at the heart of its competitive advantage.

#### CRM: Firm 11

Firm 11 is a Material Science Laboratory and a Software Design House located in the Milan area. The company is specialized in the Italian electronics market for laboratory services, professional education and consultancy, software engineering, and design services on electronic boards and components. Firm 11 has a turnover 30 employees. Thanks to its previous investments in the ERP and in a collaboration platform dedicated to internal communication, the company was able, even before the lockdown, to let its employees to work for from home, especially for the activities that could be managed remotely, such marketing, administration, buying office, design and engineering. Because part of the service provided by Firm 11 is based on the laboratory test of specific materials and electronic components, part of the employees has to go physically to the office. This preexisting (before pandemic) digital infrastructure let the company anticipate the consequences of lockdown and to experiment innovative solutions, especially for professional education. The company was able in one week to test a new set of online courses dedicated to professionals and to market them.

Due to the lockdown, the company had to radically transform the relationship with its clients. The core of this transformation was the CRM (Customer Relationships Management) platform that was already active but was underused. The pandemic pushed the company to communicate with clients in a more precise and intensive way. As the CEO of the company told us:

“… *this period has required a much more rigorous use of information than in the past...as far as customer relations are concerned, we have definitely increased the number of newsletters sent and in general we tried to use all our means to contact our clients. We have strengthened our CRM platform, because it has become essential to have quick communications with customers. We have 7000 database contacts and, trivially, having them in the database has given us a major advantage...*”*.*

Especially at the beginning of the lockdown, via CRM, the company could communicate promptly that they were open and active and could serve their clients. The company was operating in the industry of electronics that was one of the industries that could operate in the first hard Italian lockdown, but many international clients did not know if the company was open or not. Moreover, the CRM was essential to propose new services to clients like online courses for professional certification. The intensive use of CRM was a revolution in the sales process implemented by the company. Before the pandemic, the sales were based on physical events organized both in Italy and internationally, in which representatives of the companies presented the services offered. The event was anticipated by a newsletter message to clients, targeted on the base of the geographical distance of the location of the event. Usually, there were not a formal follow-up of the event, just the company expected some orders from clients. In the pandemic, this sales process clearly was not possible and the company has to use CRM to market clients one-to-one. The platform allowed the firm to increase the level of personalization of the service and the quality of the information exchanged. The company was able to improve the targeting process and to increase the quality of the follow-up thanks to the possibly of tracking all exchange of messages with clients. The results are encouraging as the CEO told us:

“*Let’s say that clients that were open during the pandemic are very happy with this type of relationship, and those that have closed and that are rebooting now I would say have very much enjoyed this way of interacting with us*”.

#### E-commerce: Firm 19

Firm 19 is specialized in the production of furniture and is based in Pordenone (close to Venice), within one of the most important Italian furniture districts. The company has a turnover of 8 million euro and 60 employees. Firm 19 was founded 20 years ago and was a traditional second tier subcontractor in the furniture supply chain of global brands like IKEA. In 2014, besides their subcontracting business, the company decided to develop their own brand, Firm 19, and to design and sell pieces of furniture such as tables, chairs, wardrobes, scaffolds, etc. directly to the final consumer. This strategic choice led to the decision to invest in ecommerce and to sell their products both in already existing marketplaces like Amazon and Ebay and directly from the company website. From a marketing point of view, the positioning of the company is primarily based on price and to the velocity of the service: in Italy they are able to deliver the product the next day of the order, internationally the process takes 3 days. Although Firm 19 is increasing the quality of the design, its competitive advantage relies on the opportunity to serve directly the final customer, cutting the costs of the intermediaries that are quite relevant in the furniture business. More importantly, the interaction with the final consumer let the company improve their learning process and increase rapidly its level of service:

“*We noticed an increase in users and therefore in searches on our site...we then developed the e-commerce channel for the final consumer...this allows us to think about the development of new types of products and make further optimizations both on the production side and on the management side and go to make improvements, but also on the site itself...*”*.*

In a matter of a few years, the B2C part of the business grew exponentially and now accounts for 70% of the total turnover of the company. Although the company did not quit subcontracting business, ecommerce is the strategic core and where the major part of the investment is concentrated.

That choice has paid off during the pandemic. The company noticed a strong increase in online orders, especially at the international level, by the final customer while the subcontracting business almost collapsed. That increase put under pressure production that has to be closed to respect the lockdown. To solve this problem, the company, on the basis of the availability of the product in the inventory, decided to establish a discount system based on the waiting time of delivery. The more the customer was willing to wait the product, the more discount. This helped in segmenting the demand on the base of their needs and expectations and helped the company to not lose orders. Talking about sales in the pandemic, the company said:

“*For your wait, we promise you a discount, so it's fine with us because we already have the product sold even before producing it, and on the other hand we have liquidity coming in that allows us to produce products that are asked for less rather than meeting other aspects, so we make a calculation within ourselves of how much we need*”.

After the lockdown, the company expected a decrease on the online sales due to the fact that stores are open and the customer could find other ways for buying furniture. Quite surprisingly, for the company, this did not happen and sales are still high. This fact has pushed the firm to increase their investment in digital technologies to improve the quality of their relationships with customers. In 2019, before pandemic, the company developed an augmented reality app that could be used to visualize the company product in the room of the client. Unfortunately, that app is not integrated with the ecommerce platform but has just a link to the site. On the basis of the results gathered during the pandemic, the company will develop soon an integration between the app and the commerce platform as well as digital marketing initiatives on social media.

## Discussion and conclusions

The study aimed at investigating, through a multiple-process analysis, how firms used digital technologies to manage markets and customers to face the challenges raised by the COVID-19 pandemic. The first step allowed us identifing what are the most important terms emerged from the word cloud analysis of the interviews collected, suggesting the priorities for firms. Such analysis highlighted the attention for customers during the pandemic, and hence the strategic role of relationships with customers to sustain business and performance. During the lockdown, the customer relationship becomes essential and firms need to improve customer relationship management systems to promote safe interactions (e.g., through online channels) and provide services to sustain business (Donthu & Gustafsson, 2020). From this point of view, our results are consistent with recent research that has stressed the advantages of servitization and how firms could invest in digital technologies to enhance service provision and new customer solutions by overcoming the physical products (Kohtamäki et al., 2020; Rapaccini et al., 2020).

In the second step of the analysis, based on cross-case comparison, we have highlighted the key role of three types of technologies, namely, videoconferencing, CRM, and e-commerce. Such technologies have been used differently within customer relationship management and they have been related to different strategic purposes. If web-based videoconferencing emerged as a key technology within the COVID-19 pandemic to overcome the physical restrictions (He et al., 2021), CRM and e-commerce become strategic technologies able to gain value in the short time (Sen & Sinha, 2011), even further prior investments implemented before the pandemic. Our findings support the consideration that today CRM strategy is always more considered “a holistic approach to managing customer relationships to create shareholder value” (Payne & Frow, 2005, p. 168).

The three case studies explored in the third part of our analysis allowed us pointing out the strategic importance of these three communication technologies in managing markets during the COVID-19 crisis. During the lockdown, those technologies played a fundamental role in reconnecting with clients. What was once taken for granted, the dialogue with clients was based on physical meetings, business fairs, social gatherings, etc. had to be rebuilt and reorganized, especially for firms with international clients. Although communications technologies were already used for several years, they played a less relevant role with respect to what emerged through the pandemic, especially in B2B. For example, video conferencing is not a recent technology and it was used for communicating with clients but was used marginally (when physical meetings were not possible) and not considered strategically relevant (Murphy & Sashi, 2018). The real value in customer relationship management was achieved during the physical meetings. In other words, the COVID-19 crisis highlighted how much the relationships with customers—especially in the B2B markets—were based on physical interactions (Kang et al., 2020).

The pandemic, and its international spread, increased the uncertainty that companies have to deal with. That uncertainty was also increased by the different regulations and lockdown rules that countries established at the same time. From our qualitative analyses, it emerges the priority of communicating with distant customers that they were open and that the business was running regularly. This was also true for companies that were closed and, nevertheless, they wanted to keep in touch with their clients communicating that they were ready to be back after the lockdown. This may seem trivial but is essential for emotionally reconnecting with clients, especially in the first days of the lockdown.

Beyond this phase of necessity, companies have to adjust their marketing and sales processes to a fully digital world due to the impossibility of physical meetings. That transformation was more complex that it may seem. If in the past, the product was the barycenter of the relationships and the service was a corollary, now servitization becomes a new strategic priority within a service-dominant logic (Lusch & Vargo, 2006). The quality of service and the capability of the firm in interacting with customers are more important than ever. The pandemic created new challenges that need to be addressed dialogically with clients and asked for new creative solutions as, for example, the discount for the delay in the delivery of the product elaborated by Firm 19. The new digital scenario helped the firm to actively involve new actors in the process of communication and to increase the quality of the service through a better involvement of the client in the design of the product directly from the early phases as we saw in the case of Firm 11. From this perspective, companies seem to use communication technologies for reaching two objectives at the same time: to increase the quality of the service offered and implement a customer-centric marketing strategy. This finding seems to confirm the literature of both servitization (Kowalkowski et al., 2017) and customer-centric marketing (Sheth et al., 2000) and brings new insights for the exploration of the combination of these two managerial practices.

Companies used communication technologies also to explore the market and find new customers. This is something more difficult to do, especially for SMEs that are used to find new clients in social gatherings (i.e. fairs). Once considered as a minimum requirement, website, social media and CRM become strategic for presenting company services and products and for searching new clients and exploring new markets. The company learned to find new clients through online initiatives and with a more systematic follow-up, as the case of firm 11 that set up a series of webinars to present their services and then via CRM used the data gathered for specific offerings. That market nurturing through digital contents and initiatives seems to be a relevant change and we expect that it will have a durable impact in the marketing strategy in the future.

In our research, we did not find evidence of an increased use of data-processing technologies (big data and AI applications) in the marketing process as proposed by the literature (Davenport et al., 2020; De Luca et al., 2020; Grandinetti, 2020; Mandelli, 2018). We think that the reason for not finding evidence is twofold. The first one is related to the fact that the pandemic (and lockdown) was an unpredictable event and caught companies totally unprepared. That phenomenon pushed firms to use technologies already at hand or easily and quickly obtainable. Big data and AI are still innovative technologies and relatively unknown to several companies. The second one depends on the complexity of the implementation of those new technological solutions. Big data and AI make data-enabled learning much more powerful than the customer insights produced in the past, but they need specific competences within the firm that take some time to develop (Bettiol et al., 2020b; Hagiu & Wright, 2020). It is understandable why, in the middle of the pandemic and to solve pressing business issues, companies tried to get out the most from existing technologies. In this sense, web-based technologies are suitable to be used in an easier and faster way because of the higher maturity. This does not mean that those companies will not use big data and AI in the near future. On the contrary, we expect that the complete digitalization of the relationships with customers could lay the ground for a fruitful application of big data and AI to the marketing process, especially for improving the customer experience in the post-COVID era (Barnes, 2020).

**Fig. 2:**
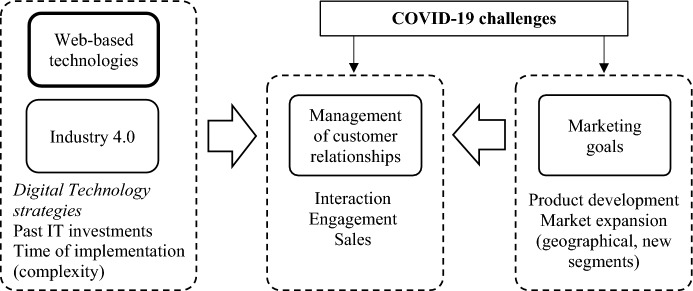
Digital connectivity in the COVID-19 scenario. *Source* Author’s elaboration

We have summarized, in the Fig. 2, the findings emerged from our analyses depicting a conceptual model that stresses the central role of the management of customers during the COVID-19 pandemic and how the firm’s digital strategy (especially the we-based technologies based on previous IT investment that require a less complex implementation and adaptation time) may allow firms to improve this customer centricity to reach marketing goals essential to face the pandemic challenges. The conceptual model allowed us to better define the contributions of the research.

### Research contributions

Theoretically, our study contributes to marketing studies exploring the impacts of digitalization on customer relationship management (Kotler et al., 2017) by expanding the discussion in the framework of crisis management. Our original study confirms the importance of exploitation of digital technologies for connecting with customers (Siggelkow & Terwiesch, 2019) but also that, especially when a crisis emerges, such as during the pandemic, such connectivity involves a plurality of technologies and not all focused on data management and efficiency. Rather, it becomes more relevant customer interaction for brand management and strengthen of customer relationships. From this perspective, we found strong evidence of the impact of web-based technologies (Abed et al., 2015) on the adoption of ecommerce practices. The pandemic has radically digitalized the relationship with costumers. Although not all transactions are closed via digital technologies, the majority of the relationship is now managed online, even in the B2B.

In addition, our study contributes to the literature on the firm’s adoption and use of digital technologies. Not only the rate and form of adoption are different in relation to the nature of the firm and of technology (Bettiol et al., 2020a; Büchi et al., 2020; Frank et al., 2019a, b; Ghobakhloo & Fathi, 2020) but also is unevenly distributed in terms of time and highly sensible to rapid change in the environment. The pandemic and lockdown boosted the adoption and increased the intensity of use of technology. That exogenous stimulus triggered a specific use of technologies, in particular in the relationship with customers, to effectively overcome physical limitations, requesting on the other side quick organizational adjustments. From this perspective, our results confirm recent studies (Papadopoulos et al., 2020) that suggests the importance of organizational ambidexterity in relation to the role played by digital technologies in the time of crisis. Both exploration (finding new solutions) and exploitation (using existing resources) were relevant in the adoption of new technologies. Business continuity and innovation are two facets of the same coin as a way to handle the challenge of the pandemic.

From a managerial point of view, our study suggests that the pandemic pushed firms, and specifically SMEs, to embrace new methods and processes in response and be reactive to the crisis, switching to new operating models focused on customers and supported by business partners. They should, therefore, be technologically ready to avoid business failure during crisis periods that may limit the physical connectivity. The use of the more mature web-based technologies may guarantee them to reach customers and continue the business activities. From the marketing point of view, COVID-19 highlighted two main key-points on which firms should focus to manage the crisis. They need to believe in the customer centricity and do the best to satisfy them and take care of them. In this regard, the new digital technologies are strategic for the effectiveness of the relationship mainly through the product and process innovation (Kim, 2020). From this perspective, we could affirm that communication is not just a mean for reaching the client with the appropriate message but is becoming the starting point of the marketing processes itself.

### Limitations and future research

We acknowledge that our study has some limitations. We analyzed only a limited number of firms, although through in-depth interviews, and coming from only one country that could influence the results we gathered. Further research should compare not only firms from different countries but also to those with a higher number of firms to verify if what we discovered is a local (Italian) phenomenon or may be generalized. Moreover, we limited the study to the first period of lockdown when firms were shocked by the pandemic and the reaction might be related also to the firm’s resilience and entrepreneur crisis management mindset. Hence, an interesting research opportunity could be to expand the time of analysis to further explore those variables that may affect the use of digital technologies for customer purposes. Indeed, future research could further investigated the framework proposed also through additional quantitative empirical studies over time, to further validate it in the post-pandemic context, taking into consideration the role had by the firm’s digital capabilities. Nevertheless, we consider our contribution relevant in the debate on the implications of COVID-19 on marketing strategies.

## Footnotes

1 We focused on SMEs because they represent the 99.7% of the Italian manufacturing companies as it is evident from https://www.istat.it/it/files/2019/12/C14.pdf
